# Regulation of the Growth‐Inhibitory Activity of Fhl1 via Interaction With Ifh1 and Crf1 at the Ribosomal Protein Gene Promoters in *Saccharomyces cerevisiae*

**DOI:** 10.1111/gtc.70109

**Published:** 2026-04-07

**Authors:** Naoki Shimamura, Koji Kasahara

**Affiliations:** ^1^ Department of Molecular Microbiology, Graduate School of Life Science Tokyo University of Agriculture Tokyo Japan

## Abstract

The transcription factor Fhl1, which binds to the promoters of the ribosomal protein genes (RPGs) in *Saccharomyces cerevisiae* , promotes transcription by recruiting the transcription activator Ifh1 under nutrient‐rich conditions. During starvation, Ifh1 dissociates from the RPG promoter, and the transcriptional repressor Crf1 binds to Fhl1 instead of Ifh1, thereby suppressing transcription. However, the lethality of the *ifh1*Δ strain was suppressed by deletion of *FHL1*, suggesting that Ifh1 has essential functions beyond transcriptional activation. Detailed analyses using various deletion mutants of Fhl1 and Ifh1 revealed that the lethality of the *ifh1*Δ strain is suppressed by mutation of the forkhead‐associated (FHA) domain, which interacts with the forkhead‐binding (FHB) domains of Ifh1 and Crf1. Inducing Fhl1‐expression in *ifh1*Δ*fhl1*Δ strain suppressed growth, indicating that promoter‐bound Fhl1 inhibits RPG transcription via an unknown mechanism. The lethality of *ifh1*Δ strain was suppressed by expression of the Ifh1 FHB domain from its native promoter or by overexpression of the corresponding domain of Crf1. These findings suggest that, while Fhl1 promotes transcription by recruiting Ifh1, dissociation of Ifh1 exposes the FHA domain, which triggers growth inhibition via an unknown mechanism. Binding to the FHA domain and neutralizing its inhibitory activity may represent a key function of Ifh1.

## Graphical abstract

Under nutrient‐rich conditions, Ifh1 recruited to the RPG promoters by the FHA domain of Fhl1 promotes growth while masking the latent inhibitory activity of Fhl1. Upon starvation or stress, dissociation of Ifh1 exposes this inhibitory activity, restricting RPG transcription and cell proliferation, whereas Crf1 functions to mitigate excessive repression mediated by Fhl1.

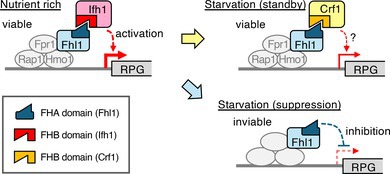

## 1 Introduction

Eukaryotic ribosomes are composed of four ribosomal RNAs (rRNAs: 25S, 18S, 5.8S, and 5S) and approximately 80 ribosomal proteins. In actively proliferating yeast cells, approximately 2000 ribosomes are newly synthesized per minute, and the transcription of rRNA (25S, 18S, and 5.8S rRNA) by Pol I accounts for approximately 60% of total cellular transcription. Furthermore, a nearly equimolar amount of 5S rRNA is transcribed by Pol III, whereas Pol II transcribes mRNAs of ribosomal protein genes (RPGs), which account for approximately 50% of total cellular mRNA (Warner 1999). Thus, synthesis of ribosomal components requires enormous energy and cellular resources, placing their regulation at the center of cellular growth, biosynthesis, and metabolism (Shore and Albert 2022). Ribosomal synthesis, which requires three distinct transcription systems, is precisely and coordinately regulated in response to nutritional signals and various stressors. Abnormalities in this regulation can disrupt protein homeostasis and, in humans, may cause numerous diseases collectively termed “ribosomopathies.” (Albert, Kos‐Braun, et al. 2019; Mills and Green 2017).

The transcriptional regulation of RPGs by Pol II is best understood in budding yeast, in which the roles of transcription factors such as Rap1, Ifh1, Fhl1, Hmo1, Fpr1, Crf1, and Sfp1 have been elucidated (Petibon et al. 2021; Shore et al. 2021), with a focus on the physical interactions and functional interdependencies among these factors. All 138 RPGs were classified into three categories (1–3) based on differences in binding to these factors (Shore et al. 2021). Most RPG promoters are classified into categories 1 and 2, both of which are characterized by binding to Rap1, the primary transcription factor for RPGs. While binding of many other factors, such as Fhl1, Ifh1, Sfp1, and Fpr1, is highly similar between the two categories, the major difference is that the HMG protein Hmo1 binds to category 1 RPGs but not to category 2 RPGs (Knight et al. 2014; Kasahara et al. 2007). Fhl1 binding to nearly all category 1 RPGs depends on Hmo1. However, Fhl1 binding to category 2 RPGs does not depend on Hmo1, and binding to a subset of category 2 RPGs depends solely on Fpr1 (Kasahara 2021). However, many RPGs do not depend on either Hmo1 or Fpr1, and the mechanism underlying this binding remains largely unknown. In Category 3 RPGs, Abf1 binds instead of Rap1, and binding of Fpr1, Fhl1, and Ifh1 is absent or extremely low (Bosio, Fermi, and Dieci 2017; Bosio, Fermi, Spagnoli, et al. 2017; Zencir et al. 2020). Fhl1 binds at a specific distance from the Rap1 binding site and is regulated by Rap1, Hmo1, and Fpr1 (Knight et al. 2014; Reja et al. 2015; Kasahara et al. 2020). Although Fhl1 contains the FH domain, a DNA‐binding motif, and the vicinity of the Fhl1 binding site includes a consensus sequence termed the IFHL motif (Wade et al. 2004; Hall et al. 2006; Zhao et al. 2006; Knight et al. 2014), the roles of these elements in Fhl1 promoter binding remain unclear. Sfp1 binds not only to RPGs but also to promoters of RiBi (ribosomal biosynthesis) genes, whose products are involved in ribosome synthesis rather than in ribosomes themselves (Jorgensen et al. 2002; Jorgensen et al. 2004; Fingerman et al. 2003; Albert, Tomassetti, et al. 2019). However, the binding modes of Sfp1 to these two targets differ. While Sfp1 binding to the latter promoters, detected by ChEC methods, possibly occurs via direct binding to a specific consensus sequence, binding to the former promoters, detected by ChIP methods, may depend on Fhl1 and Ifh1 (Albert, Tomassetti, et al. 2019). However, their specific roles in transcription of these promoters remain unclear.

Ifh1 is considered a major transcriptional activator in RPG transcription and, together with Rap1, is essential for growth. RPG transcription is dynamically regulated in response to nutritional conditions, changes in Pol I activity (Laferte et al. 2006), and various stressors, with Ifh1 serving as the primary target of signals from these regulatory events (Albert, Kos‐Braun, et al. 2019). Ifh1 binds to RPG promoters under optimal nutritional conditions via an interaction between the FHA domain of Fhl1 and the FHB domain of Ifh1. While the addition of rapamycin, which mimics starvation conditions, causes Ifh1 to dissociate from promoters, the binding of Fhl1 to RPG promoters remains largely unchanged (Martin et al. 2004; Schawalder et al. 2004; Wade et al. 2004; Rudra et al. 2005). At this point, Crf1, a paralog of Ifh1, is thought to translocate to the nucleus, bind to the FHA domain instead of Ifh1, and suppress transcription (Martin et al. 2004).

Phosphorylation of Ifh1 by Sch9 or CK2 may regulate the Fhl1–Ifh1 interaction (Albert et al. 2016) (Kim and Hahn 2016). The N‐terminus of Ifh1 is acetylated by Gcn5, a histone acetyltransferase, and deacetylated by histone deacetylases such as Sir2 or Hst2 (Downey et al. 2013). The addition of rapamycin rapidly reduces acetylation, and acetylation by Gcn5 negatively regulates Ifh1 activity at promoters and Ifh1‐mediated transcriptional activation. While rapamycin‐induced dissociation of Ifh1 from the promoter occurs via a Utp22‐independent mechanism in the short term, long‐term stable dissociation involves sequestration of Ifh1 into the CURI complex, which is composed of CK2, Utp22, Rrp7, and Ifh1 through its C‐terminal domain (Rudra et al. 2007; Rudra and Warner 2016; Albert et al. 2016; Shore et al. 2021). This interaction may contribute to the cooperative inhibition of Pol I and Pol II activity to efficiently regulate production of ribosomal components, rRNA, and ribosomal proteins. Furthermore, unassembled ribosomal proteins, which increase owing to rRNA transcription inhibition, abnormalities in ribosomal protein synthesis, or other stresses, form aggregates. Incorporation of Ifh1 into these aggregates results in decreased RPG transcription. This cellular response, termed RASTR (Ribosome Assembly Stress Response), maintains protein homeostasis and protects cells from proteotoxic stress (Albert, Kos‐Braun, et al. 2019; Shore et al. 2021). Furthermore, recent studies revealed that Stm1, a ribosomal dormancy factor, activates Ifh1 by interacting with the transcription activation domain of Ifh1 via its own C‐terminal intrinsically disordered region (IDR), thereby promoting RPG transcription, whereas during nutrient starvation, phosphorylation of the Stm1 C‐terminal IDR disrupts this interaction with Ifh1, thereby suppressing RPG transcription (Bianco et al. 2025). Thus, Ifh1 serves as a signaling hub to achieve appropriate ribosome biosynthesis in response to various intracellular and extracellular environments. Deletion of *IFH1* causes growth arrest, indicating that it plays a central role in ribosomal synthesis. Curiously, however, the lethality of *IFH1* gene knockout strains (*ifh1*Δ) is suppressed by deletion of Fhl1, a scaffold for Ifh1 on RPG promoters (Cherel and Thuriaux 1995; Rudra et al. 2005), or by overexpression of the competitive factor of Ifh1, Crf1 (Zencir et al. 2020). Furthermore, recent findings showing that deletion of Crf1 reduces the transcription of target genes such as *HMO1* and *UTP22* in addition to RPGs (Kumar et al. 2024), suggest that Crf1 is not a transcriptional repressor for certain genes, as previously thought, but rather a weaker transcription activator compared to the strong activator Ifh1 (Zencir et al. 2020; Kumar et al. 2024). These findings suggest that the long‐standing model involving Fhl1, Ifh1, and Crf1, in response to various environments requires modification.

In this study, we performed a more detailed analysis of the mechanism underlying suppression of lethality in the *ifh1*Δ strain. Deletion of the entire *FHL1* gene, as well as deletion of or point mutations in the FHA domain, permitted growth of the *ifh1*Δ strain. Furthermore, expression of the FHB domain of Ifh1 from its endogenous promoter, and even overexpression of the FHB domain of Crf1, also supported growth of the *ifh1*Δ strain.

These results strongly suggest that the growth defect of *ifh1*Δ strain arises from an unknown growth‐inhibitory activity of the FHA domain, which is exposed by the Ifh1 deletion. In other words, the essential role of Ifh1 in growth is not the promotion of RPG transcription but the suppression of this growth‐inhibitory activity.

## 2 Results

### 2.1 Essential Function of Ifh1 for Cell Viability Can Be Bypassed by Deletion of *FHL1*

Ifh1 is believed to play a central role in transcriptional activation of RPGs, and a deletion mutant of *IFH1* (*ifh1*Δ) is lethal. Under nutrient‐rich conditions, Ifh1 binds to RPG promoters in a manner dependent on Fhl1 (Martin et al. 2004; Schawalder et al. 2004; Wade et al. 2004; Rudra et al. 2005). Although deletion of *FHL1* almost abolishes Ifh1 binding to RPG promoters, the *fhl1*Δ strain is viable. Intriguingly, a previous study demonstrated that the *ifh1*Δ strain was viable in a *fhl1*Δ background (i.e., *ifh1*Δ*fhl1*Δ strain was viable) (Cherel and Thuriaux 1995; Rudra et al. 2005). At the start of this study, we confirmed this synthetic effect to investigate the mechanism underlying suppression of lethality of the *ifh1*Δ strain by deletion of *FHL1*.

**FIGURE 1:**
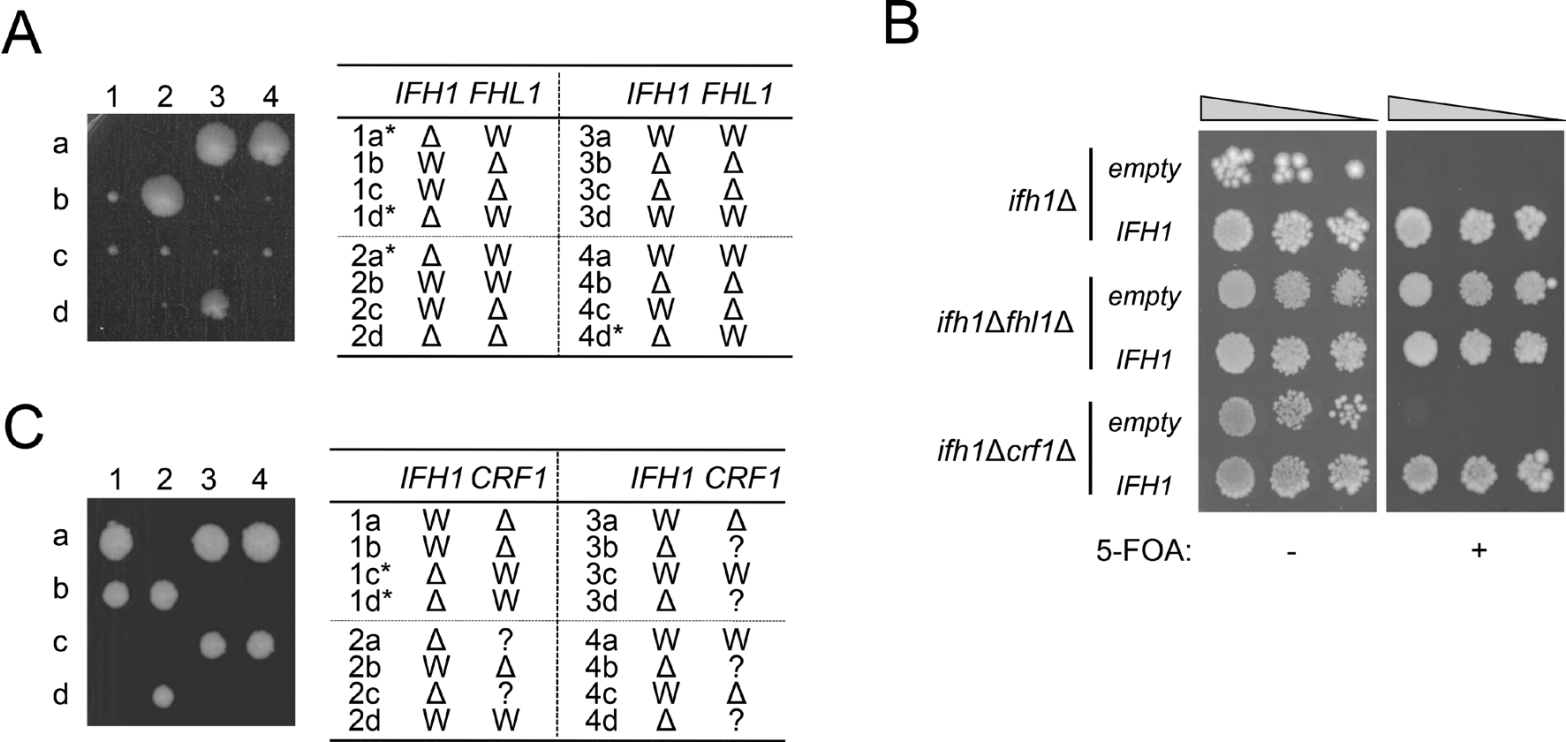
Genetic interaction between *IFH1* and *FHL1* or *CRF1*. (A) Effect of *ifh1*Δ and/or *ifh1*Δ*fhl1*Δ on growth. A diploid strain (YKK1538: *FHL1*/*fhl1*Δ *IFH1*/*ifh1*Δ) was subjected to tetrad dissection analysis. Images were acquired 7 days after tetrad dissection. Representative samples of tetrads are numbered 1–4, and four segregants from each tetrad are labeled a–d (one or two segregants were inviable in tetrads 1, 2, and 4). The genotype of each viable segregant was examined using polymerase chain reaction (PCR) and is indicated in the table to the right of the image. The genotypes of inviable segregants marked with an asterisk (*) were inferred from those of other viable cells. (B) *ifh1*Δ, *ifh1*Δ*fhl1*Δ and *ifh1*Δ*crf1*Δ strains harboring the *IFH1*/*URA3* plasmid were transformed with the *IFH1*/*LEU2* plasmid or a *LEU2*‐empty plasmid. These transformants were spotted on medium with or without 5‐fluoroorotic acid (5‐FOA) at three dilutions and grown for 5 days at 30°C. (C) Effect of *ifh1*Δ and/or *ifh1*Δ*crf1*Δ on growth. A diploid strain (YKK1539: *CRF1*/*crf1*Δ *IFH1*/*ifh1*Δ) was subjected to tetrad dissection analysis as described in (A). Status of *CRF1* gene (*crf1*Δ or *CRF1*) of inviable segregants with asterisk (*) could not be specified and was therefore described as “?”

First, a diploid strain in which one of the two *FHL1* alleles and one of the two *IFH1* alleles was deleted (*FHL1*/*fhl1*Δ *IFH1*/*ifh1*Δ) was sporulated and dissected. The genotypes of all viable colonies were examined using colony polymerase chain reaction (PCR). The results indicated that viable colonies were WT, *fhl1*Δ, or *ifh1*Δ*fhl1*Δ, whereas the *ifh1*Δ strain was not viable (Figure 1A). Next, *ifh1*Δ and *ifh1*Δ*fhl1*Δ strains harboring *IFH1* on a *URA3*‐marker plasmid (hereafter, *IFH1*/*URA3* plasmid) were established. These strains were transformed with an *IFH1*/*LEU2* plasmid or *LEU2*‐empty plasmid. Transformants were spotted on a medium plate containing 5‐FOA (hereafter, 5‐FOA plate). The *ifh1*Δ*fhl1*Δ strain grew on the 5‐FOA plate by losing the *IFH1*/*URA3* plasmid, regardless of whether it possessed *IFH1/LEU2* plasmid. In contrast, the *ifh1*Δ strain grew on the 5‐FOA plate only in the presence of the *IFH1/LEU2* plasmid (Figure 1B). These results indicate that the deletion of *IFH1* is not lethal in an *fhl1*Δ background, consistent with previous reports. These observations suggest that Fhl1 negatively affects growth in the absence of Ifh1, although the underlying mechanisms remain unknown. Ifh1 binds to RPG promoters via interaction with Fhl1, thereby promoting RPG transcription under nutrient‐rich conditions. In contrast, under starvation conditions, Ifh1 dissociates from RPG promoters, and Crf1 binds to Fhl1, which is thought to repress RPG transcription (Martin et al. 2004). We hypothesized that Crf1 preferentially binds to Fhl1 and represses the transcription of RPGs in the *ifh1*Δ strain even under nutrient‐rich conditions, thereby prohibiting growth of the *ifh1*Δ strain. To test this hypothesis, we examined the genetic interactions between the *ifh1*Δ and *crf1*Δ. First, a diploid strain in which one of the two *CRF1* alleles and one of the two *IFH1* alleles was deleted (*CRF1*/*crf1*Δ *IFH1*/*ifh1*Δ) was sporulated and dissected. The results clearly indicated that only two colonies could grow in all sets of tetrads, and that all viable colonies contained the *IFH1* gene (Figure 1C), suggesting that all *ifh1*Δ strains, regardless of whether they possessed *CRF1*, were inviable. Next, *ifh1*Δ and *ifh1*Δ*crf1*Δ strains harboring the *IFH1*/*URA3* plasmid were established. When these strains were transformed with the *IFH1*/*LEU2* plasmid, both strains grew on the 5‐FOA plate, whereas both strains transformed with the *LEU2*‐empty plasmid did not grow on the same medium (Figure 1B). These results strongly suggest that the lethality of *ifh1*Δ could not be suppressed by deletion of *CRF1*.

### 2.2 FHA Domain and a C‐Terminal Region of Fhl1 Are Responsible for Growth Inhibition of *ifh1*Δ Strain

These results suggest that Fhl1 inhibits yeast growth in an *ifh1*Δ background. AlphaFold 3 predicts three domains within Fhl1: two previously characterized domains, the FHA and FH domains, and a third domain whose function remains uncharacterized (Domain 3; Figure S1A). To determine which region is responsible for the growth‐inhibitory activity of Fhl1, a series of *FHL1* deletion mutant genes were constructed on a *LEU2*‐marker plasmid. These plasmids were introduced into the *fhl1*Δ strain, which is viable but exhibits markedly slower growth, and the resulting transformants were spotted on SD‐Leu medium (Figure S1B). As expected from previous reports, deletion (Δ300–374) or point mutation (S325R) in the FHA domain (forkhead‐associated domain), which is known to be deficient in interaction with Ifh1, significantly diminished growth complementation of the *fhl1*Δ strain. In addition, deletion of an uncharacterized region within the C‐terminal half of Fhl1 (Δ572–662) exhibited a moderate inhibitory effect on the growth of the *fhl1*Δ strain (Figure S1B). The amount of mutant Fhl1 protein expressed here was examined by immunoblotting. As demonstrated in Figure S1C, deletion of the FHA domain (Δ300–374) almost eliminated Fhl1 protein, and similarly, the S325R mutation significantly decreased Fhl1 protein (Figure S1C). In contrast, the G303A mutation in the FHA domain, which is also deficient in interaction with Ifh1 (Martin et al. 2004), exhibited a rather mild effect on the stability of the Fhl1 protein (Figure S1C). Thus, in our strain background, deletion or mutation of the FHA domain significantly affected the amount of Fhl1 protein. In contrast, in a previous experiment by Rudra et al., the effect of deletion of the FHA domain or the S325R mutation on the amount of Fhl1 protein was only moderate, although these mutant cells exhibited significantly slower growth (Rudra et al. 2005). While Rudra et al. expressed mutant Fhl1 in W303‐1A derived *fhl1*Δ strains, we conducted this experiment using the BY4741 strain background. To examine whether the difference in the amount of those Fhl1 mutant proteins is due to different strain backgrounds, we constructed *fhl1*Δ strain in the W303‐1A background, and Δ300–374 and S325R mutant Fhl1 were expressed in this *fhl1*Δ strain. These Fhl1 mutant proteins showed very slight expression in the W303‐1A background, as observed in the BY4741 background (Figure S2A). At present, the reason for the difference in the expression of mutant Fhl1 between the study by Rudra et al. and ours remains unclear.

**FIGURE 2:**
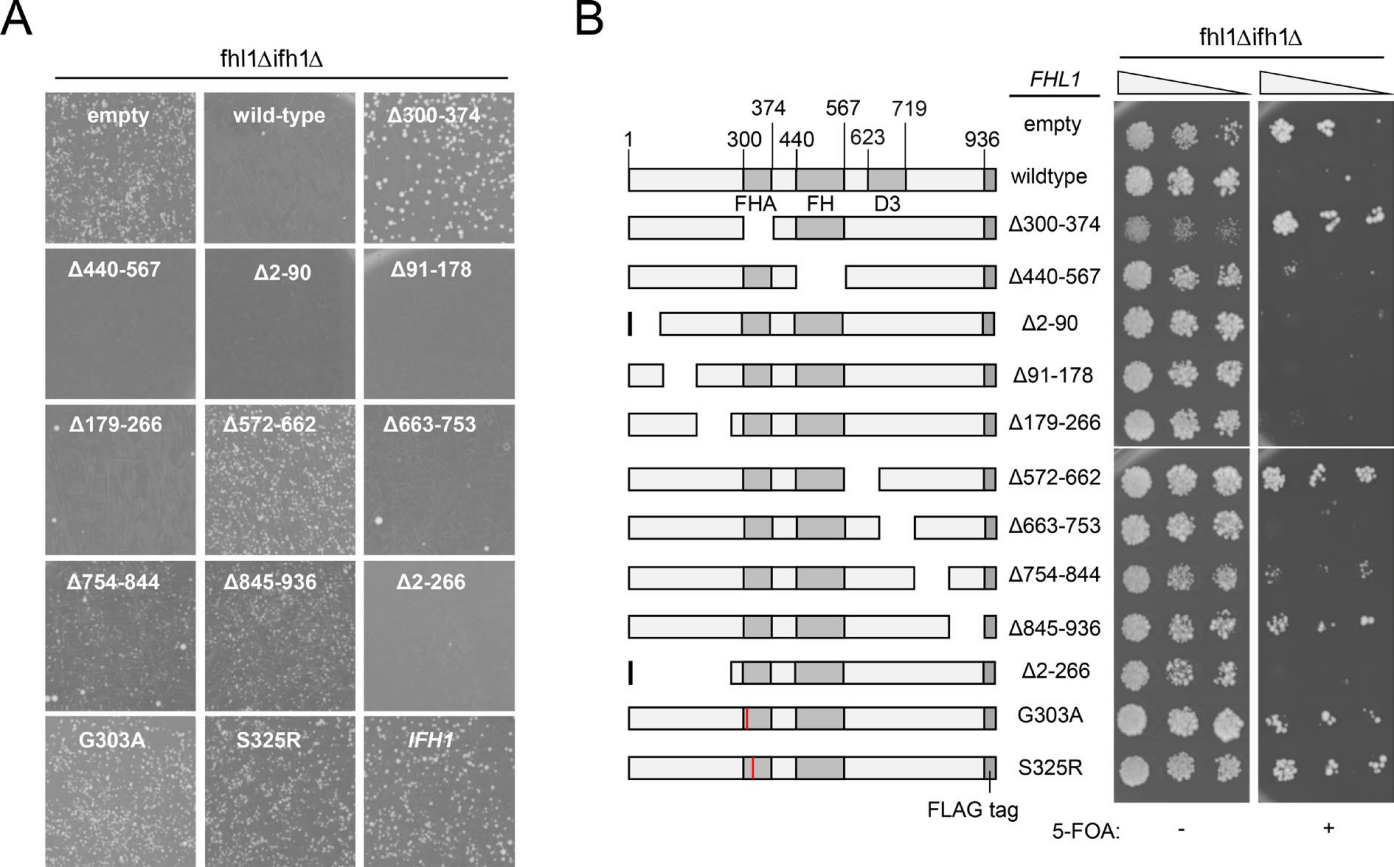
Effect of deletion or mutation of Fhl1 protein on its growth‐inhibitory activity in the background of *ifh1*Δ. (A) The *ifh1*Δ*fhl1*Δ strain was transformed with plasmids expressing Fhl1 proteins with various deletions or point mutations, spread on SD–Leu plates, and grown for 10 days at 30°C. (B) The *ifh1*Δ*fhl1*Δ strain harboring the *IFH1/URA3* plasmid was transformed with the same plasmids used in (A). The transformants were then spotted on medium with or without 5‐FOA at three dilutions and grown for 7 days at 30°C.

Next, these *FHL1* mutants were transformed into *ifh1*Δ*fhl1*Δ strain, which was viable, as described above. As expected, almost no colonies appeared when the wild‐type *FHL1* was transformed into *ifh1*Δ*fhl1*Δ strain (Figure 2A). In addition, transformant of *FHL1* mutants with deletion of N‐terminus (Δ2–90, Δ91–178, Δ179–266, and Δ2–266) and FH domain (forkhead domain: Δ440–567) exhibited a similar effect to that of wild‐type. In contrast, transformant of *FHL1* mutants with deletion of FHA domain (forkhead associated domain: Δ300–374) or point mutations in FHA domain (S325R and G303A) that are reported to abrogate interaction with FHB domain of Ifh1 could form viable colonies (Figure 2A). In addition, several regions scattered on the C‐terminal side of the FH domain (Δ572–662, Δ754–844, and Δ845–936) could form viable colonies (Figure 2A). These results suggest that these regions are involved in growth inhibition by Fhl1 in the background of *ifh1*Δ.

Inhibitory effects on growth were examined using another method. The *ifh1*Δ*fhl1*Δ strain harboring *IFH1*/*URA3* plasmid was transformed with plasmids expressing mutant *FHL1* genes used in the above experiment, and the transformants were spotted on the 5‐FOA plate. The results indicated that wild‐type and mutant strains with deletion of the FH domain and N‐terminal regions (Δ2–90, Δ91–178, Δ179–266, and Δ2–266) could not form colonies on the 5‐FOA plate, possibly because these Fhl1 proteins retained growth‐inhibitory activity in the *ifh1*Δ strain (Figure 2B). On the contrary, deletion or point mutation (S325R and G303A) of the FHA domain and C‐terminal regions (except for Δ663–753) could form colonies similarly to the empty plasmid (Figure 2B), suggesting that these mutants were defective in growth‐inhibitory activity in the *ifh1*Δ strain.

As described above, ΔFHA (Δ300–374) or S325R mutant Fhl1 proteins showed very slight expression in our strain backgrounds (BY4741 and W303‐1A, Figures S1C, lanes 2 and 3, and S2A, lanes 5 and 9). Therefore, the effect of these mutations on the improved growth of the *ifh1*Δ*fhl1*Δ strain might be due to loss of the Fhl1 protein itself, but not to loss of specific inhibitory activity. Therefore, we constructed high‐copy plasmids expressing these mutant Fhl1 proteins from their own promoters and used these plasmids for the same experiments conducted in Figure 2 to examine the effect of mutant Fhl1 on the growth of the *ifh1*Δ*fhl1*Δ strain. Immunoblotting analysis indicated that the levels of these Fhl1 proteins expressed in high‐copy plasmids were comparable to those of wild‐type Fhl1 expressed in the low‐copy plasmid (Figure S2A, lanes 7 and 11). When these plasmids were transformed into the *ifh1*Δ*fhl1*Δ strain, viable colonies were formed on the plate, as observed above, using a low‐copy plasmid (Figure S2B). Furthermore, the *ifh1*Δ*fhl1*Δ strain harboring *IFH1*/*URA3* plasmid was transformed with the high‐copy plasmids expressing ΔFHA, S325R, ΔFH, and wild‐type Fhl1 protein, and these transformants were spotted on the medium with or without 5‐FOA. The result indicated that the *ifh1*Δ*fhl1*Δ strain expressing ΔFHA or S325R mutant Fhl1 proteins could form colonies on the 5‐FOA plates (Figure S2C), as observed above, using a low‐copy plasmid (Figure 2B). These results strongly suggest that the FHA domain inhibits growth of the *ifh1*Δ strain.

### 2.3 Fhl1 Represses Transcription of RPGs in the *ifh1*Δ Strain, Thereby Suppressing Growth of Yeast

**FIGURE 3:**
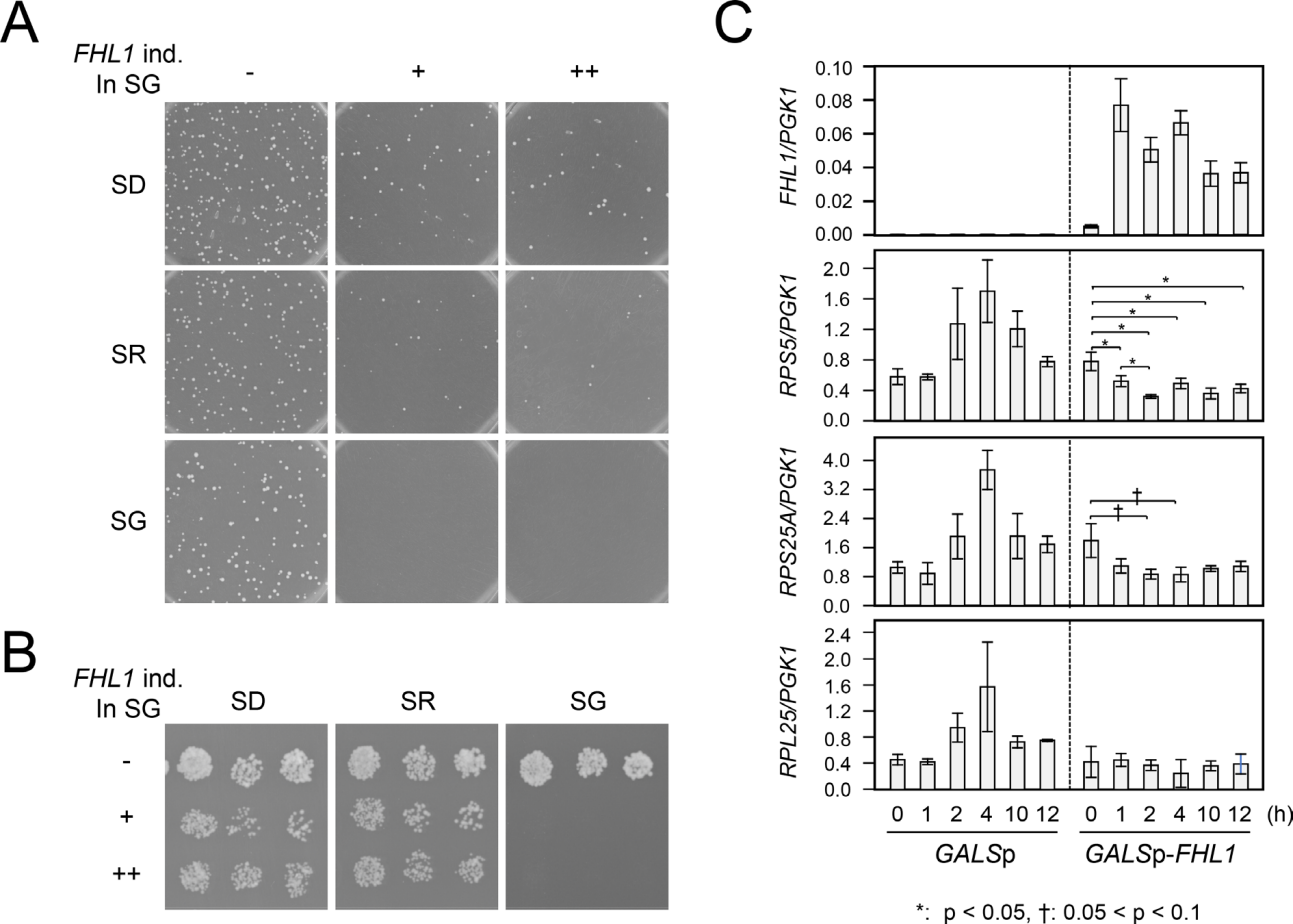
Effect of induction of Fhl1 on the growth and transcription of ribosomal protein genes (RPGs) of the *ifh1*Δ*fhl1*Δ strain. (A) The *ifh1*Δ*fhl1*Δ strain was transformed with the low‐ or high‐copy plasmids expressing *FHL1* under galactose‐inducible promoter (*GALS*p) and was spread on synthetic medium plates containing 2% glucose (SD), 2% raffinose (SR), or 2% galactose (SG) as the sole carbon source, and grown for 10 days at 30°C. The symbols “−,” “+,” and “++” on the images indicate the strength of the *FHL1* induction by galactose (*GALS*p empty plasmid, *GALS*p‐*FHL1*/low‐copy plasmid, and *GALS*p‐*FHL1*/high‐copy plasmid, respectively). (B) The transformant used in (A) were spotted on the SD, SR, and SG medium plate, and grown for 8 days at 30°C. (C) The *ifh1*Δ*fhl1*Δ strains harboring empty plasmid or *GALS*p‐*FHL1*/low‐copy plasmid used in (A) were cultured in 10 mL of SR liquid medium for 24 h at 30°C. The cultures were then inoculated into 300 mL of SR liquid medium and grown for an additional 24 h. Subsequently, 50 mL of each culture was inoculated into six equal volumes of SG medium containing 4% galactose to induce *FHL* *1* expression. Total RNA was prepared from culture broth at 1, 2, 4, 10, and 12 h after initiation of induction. Reverse transcription quantitative polymerase chain reaction (RT‐qPCR) was conducted using prepared RNA to quantify the mRNA of several genes (*FHL1*, *RPS5*, *RPL25*, *RPS25*A, and *PGK1*) and indicated as bar chart. Each experiment was conducted in triplicate, and the average and SD for the ratio of amounts of mRNAs of RPGs versus *PGK1* was calculated. For galactose‐induced strains, the t‐test was used to examine significant differences in RPG mRNA levels before and after induction.

For the first step to elucidate this growth inhibition mechanism, we examined the effect of Fhl1 induction on the transcription of RPGs in the *ifh1*Δ*fhl1*Δ strain. The *ifh1*Δ*fhl1*Δ strain was transformed with plasmids that drive the transcription of *FHL1* under the control of *GALS* promoter (*GALS*p, a truncated *GAL1* promoter) and spread on synthetic medium plates containing glucose (SD), raffinose (SR), or galactose (SG) as the sole carbon source. The result indicated that the *ifh1*Δ*fhl1*Δ strain possessing *GALS*p‐*FHL1* constructs grew on SD and SR medium plates but did not grow on SG plates. In contrast, the *ifh1*Δ*fhl1*Δ strain possessing the empty vector containing only *GALS* promoter grew on any of the medium plates tested here (Figure 3A). Growth of these transformants was examined by spotting them on media containing different carbon sources. Consistent with the results of the initial transformation, the *ifh1*Δ*fhl1*Δ strain containing *GALS*p‐*FHL1* constructs did not grow specifically on the SG plate (Figure 3B).

The effect of the induction of *FHL1* on transcription of RPGs was examined using these transformants. After pre‐culture in SR liquid medium, the cells were transferred into SG medium according to the procedure described in the Experimental procedures section and/or Figure legend. At 1, 2, 4, 10, and 12 h after initiating *FHL1* induction by galactose, we examined the effects of Fhl1 induction on transcription. The mRNA levels of RPGs (*RPS5* and *RPS25*A) significantly decreased upon induction of *FHL1*, compared to a non‐RPG, *PGK1* (Figure 3C). This result indicates that Fhl1 suppressed the growth of *ifh1*Δ*fhl1*Δ strain by inhibiting RPG transcription via an unknown mechanism, although the involvement of the FHA domain in this inhibitory effect has not been directly demonstrated. The apparent weaker reduction of *RPL25* than *RPS5* and *RPS25A* likely reflects its already reduced expression during preculture in SR medium. Low basal *FHL1* expression may have suppressed *RPL25* more strongly than *RPS5* or *RPS25A*, thereby limiting the extent of further decrease upon galactose induction.

Notably, the increase in RPG mRNA in the *GALS*p control (without *FHL1*) was observed after the shift to SG medium (Figure 3C). In this experiment, cells were precultured in raffinose medium, where growth is suboptimal compared with galactose, and had reached stationary phase before dilution. Dilution into fresh galactose medium likely triggered re‐entry into logarithmic growth. Thus, the observed increase in mRNA of RPGs in the control strain likely reflects a general carbon‐source and growth‐phase effect.

### 2.4 FHB Domain of Ifh1 Is Sufficient to Enable the Growth of *ifh1*Δ Strain

**FIGURE 4:**
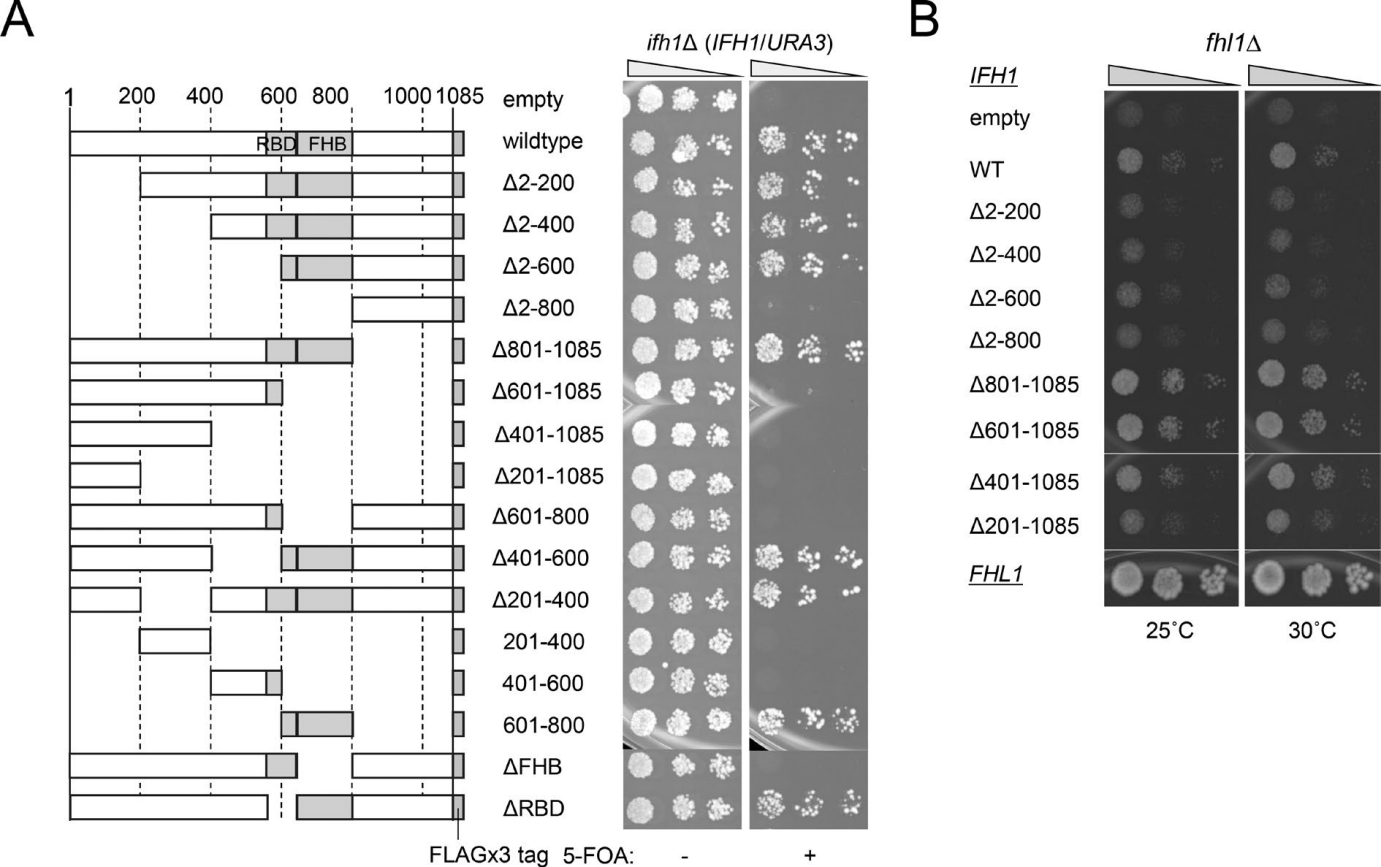
Functional analysis of Ifh1 mutants by overexpression in *ifh1*Δ and *fhl1*Δ strains. (A) Effect of overexpression of various Ifh1 mutant proteins on growth of the *ifh1*Δ strain. High‐copy *LEU2*‐marker plasmids expressing various Ifh1 deletion mutant proteins from their own promoter were transformed into the *ifh1*Δ strain harboring the *IFH1/URA3* plasmid. The transformants were spotted on medium with or without 5‐FOA at three dilutions and grown for 3 days at 30°C. (B) Effect of overexpression of Ifh1 mutants on the growth of the *fhl1*Δ strain. Plasmids expressing various Ifh1 deletion proteins used in Figure 4 were transformed into the *fhl1*Δ strain. These transformants were spotted on the SD‐Leu medium at three dilutions, and grown for 7 days at 25°C, or 5 days at 30°C.

As reported previously, Ifh1 is recruited to RPG promoters via the interaction between its FHB domain and the FHA domain of Fhl1, thereby promoting the transcription of RPGs. This transcriptional activation is considered an essential function of Ifh1 in growth. However, the rather puzzling result is that the lethality of the *ifh1*Δ strain is suppressed by the deletion or mutation of the FHA domain of *FHL1* that abolishes Ifh1 recruitment. To interpret this observation, we hypothesized that the growth‐inhibitory activity of the FHA domain was masked by Ifh1 binding. In other words, the essential function of Ifh1 for growth may not be to promote transcription after recruitment via interaction with the FHA domain but rather to suppress the growth‐inhibitory activity of Fhl1. To examine this idea, high‐copy plasmids expressing various Ifh1 deletion mutant proteins were transformed into the *ifh1*Δ strain harboring the *IFH1*/*URA3* plasmid, and these transformants were spotted on the 5‐FOA plate. The results clearly indicated that mutant proteins lacking the N‐terminus (up to 600aa), the C‐terminus (beyond 800aa), or both could enable the growth of the *ifh1*Δ strain on the 5‐FOA plate nearly as effectively as the wild‐type Ifh1 (Figure 4A). Remarkably, the small region of 601–800aa was sufficient to enable the growth of the *ifh1*Δ strain to a level comparable to wild‐type when expressed from its own promoter on a high‐copy plasmid (Figure 4A), or even when expressed on a low‐copy plasmid (Figure S3). At present, we restrict the smallest region of Ifh1 that can enable the growth of the *ifh1*Δ strain to 678–788aa (Figure S3). This region is the core of the FHB domain and is required for interaction with the FHA domain. To examine whether the binding activity of the FHB domain to Fhl1 is indispensable for the growth of the *ifh1*Δ strain, Thr681 or Ser680/Thr681 in the Ifh1, whose phosphorylation is important for interaction with Fhl1, were replaced with alanine (T681A and ST(680,681)AA) in the full‐length, 601–800aa region, or smallest FHB domains (678–788aa). As a result, substitution of these amino acids abolished the rescue activity of the smallest FHB domain and markedly impaired the rescue activity of the 601–800aa region (Figure S3).

A previous study showed that two bipartite regions in Ifh1, AD1 (716–822aa) and AD2 (911–1030aa), exhibit transcriptional activation activity for the reporter gene when fused with the DNA‐binding domain (Zhong and Melcher 2010). In addition, a recent study showed that the region overlapping the AD (703–811aa) of Ifh1 interacts with Stm1, thereby regulating the transcription of RPGs (Bianco et al. 2025). Although the smallest FHB domain described above (678–788aa) partially overlaps with the activation domain of Ifh1 described in these papers, the result that mutation of Ser680 and Thr681, which are outside this region, resulted in loss of activity to enable the growth of the *ifh1*Δ strain (Figure S3) suggests that suppressing the growth‐inhibitory activity of FHA, rather than FHB‐mediated transcriptional activation, is an essential function of Ifh1 for growth.

### 2.5 Ifh1 May Contribute to Yeast Growth Through a Mechanism Independent of Transcriptional Activation

In previous studies, suppression of Ifh1 expression under the control of the *GAL1* promoter, as well as rapamycin‐induced dissociation of Ifh1 from RPG promoters, was associated with decreased transcription of RPGs, leading to the view that Ifh1 promotes RPG transcription (Martin et al. 2004; Wade et al. 2004; Schawalder et al. 2004; Rudra et al. 2005). However, our findings suggest that loss of Ifh1 may simultaneously unmask Fhl1‐mediated repression of RPG transcription. Therefore, it is difficult to determine whether Ifh1 acts as a direct transcriptional activator or instead promotes transcription indirectly by relieving Fhl1‐dependent repression. As previously reported, overexpression of *IFH1* partially suppressed the slow growth of the *fhl1*Δ strain (Cherel and Thuriaux 1995). In the *fhl1*Δ background, Ifh1 no longer needs to mask the growth‐inhibitory effect mediated by the FHA domain of Fhl1, suggesting that growth promotion in the *fhl1*Δ strain occurs through a different mechanism, such as transcriptional activation. To investigate this mechanism, we first overexpressed a series of Ifh1 deletion mutants in the *fhl1*Δ strain and examined which regions of Ifh1 are required for improved growth. Deletion of the N‐terminal regions containing 2–200aa (Δ2–200, Δ2–400, Δ2–600, and Δ2–800) diminished the suppression of slow growth in the *fhl1*Δ strain (Figure 4B). In contrast, C‐terminal deletion mutants exhibited growth‐promoting effects equivalent to (or more effective than) wild‐type. This result suggests that the FHB domain and the C‐terminal region containing AD1 and AD2 of Ifh1 are not essential for its growth‐promoting activity, at least in this context.

Next, we examined the effects of overexpressing the above Ifh1 deletion mutants on expression of RPGs. Unexpectedly, the Ifh1 mutants that promoted growth, as well as wild‐type Ifh1, showed little or no stimulation of RPG transcription compared with other mutants or the empty vector that failed to promote growth (Figure S4). Moreover, no clear correlation was observed between RPG transcription and growth recovery. These results suggest that Ifh1 may promote cell growth through a mechanism that is not necessarily dependent on its ability to stimulate RPG transcription under these conditions. Consistent with this possibility, at least in this experimental system using *fhl1*Δ cells as the host, Ifh1 did not exhibit detectable activity in stimulating RPG transcription.

### 2.6 FHB Domain of Crf1 Is Sufficient to Enable the Growth of *ifh1*Δ Strain

**FIGURE 5:**
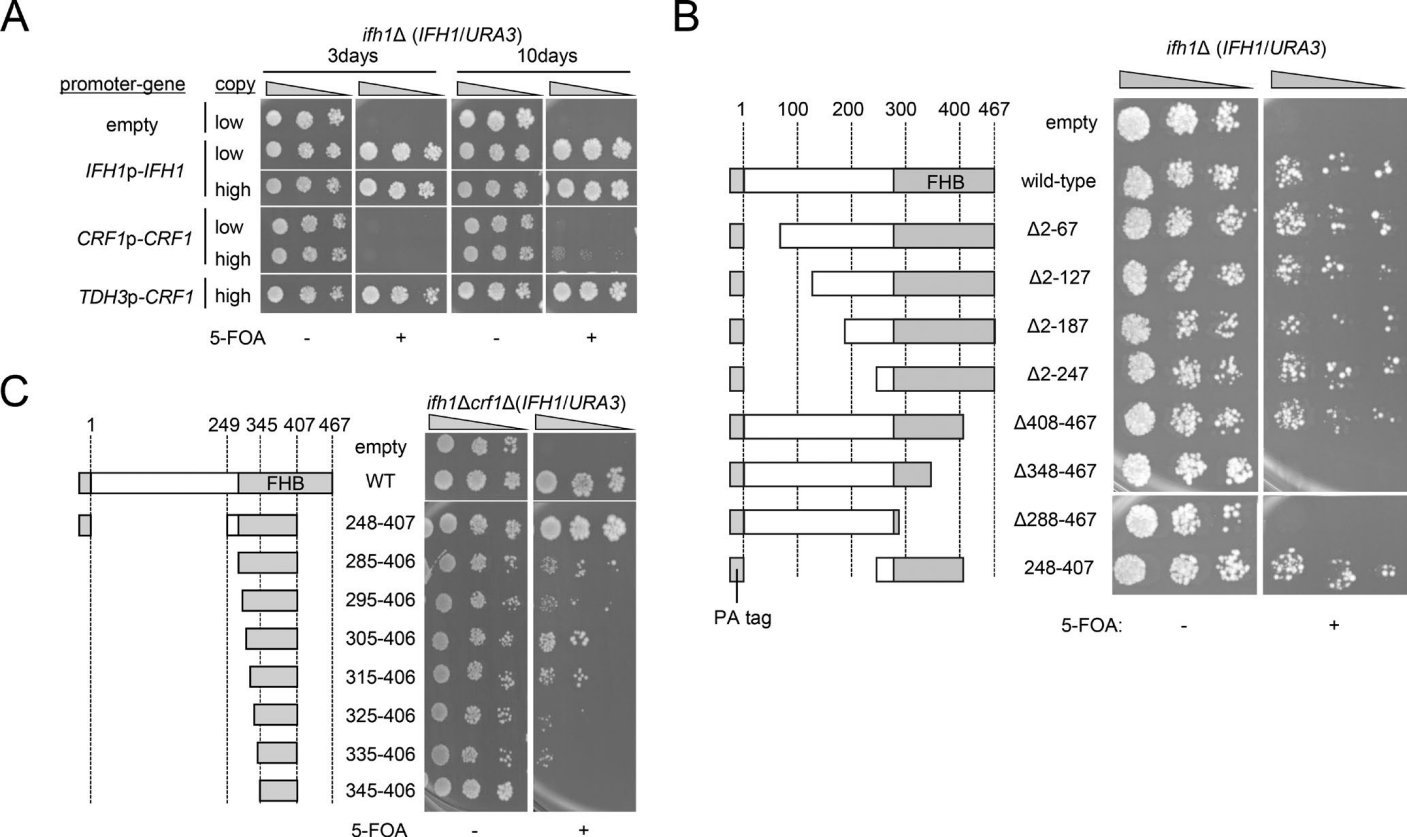
Complementation of growth of the *ifh1*Δ strain by overexpression of various Crf1 mutant proteins. (A) Effect of the overexpression of Crf1 on growth of the *ifh1*Δ strain. The plasmids expressing Crf1 from its own promoter (*CRF1*p) or *TDH3* promoter (*TDH3*p) were constructed using low‐copy and high‐copy vectors. These plasmids, in addition to low‐ or high‐copy plasmids expressing *Ifh1* from its own promoter (*IFH1*p), were transformed into the *ifh1*Δ strain harboring *IFH1/URA3* plasmid. These transformants were spotted on the medium with or without 5‐FOA at three dilutions and grown at 30°C. The images were acquired 3 or 10 days after spotting. (B) Plasmids expressing various Crf1 deletion mutant proteins from the *TDH3* promoter, which were constructed using high‐copy vectors, were transformed into the *ifh1*Δ strain harboring *IFH1/URA3* plasmid. These transformants were spotted on the medium with or without 5‐FOA as described in (A). The dark gray and blue squares represent the FHB domain and PA tag, respectively. (C) Plasmids expressing the 248–407aa region of Crf1 or its truncated version (285–406aa, 295–406aa, 305–406aa, 315–406aa, 325–406aa, 335–406aa, and 345–406aa) were transformed into the *ifh1*Δ strain, and their growth‐complementing activity was examined as described in (A) for 10 days.

The observations that overexpression of the FHB domain of Ifh1 enable growth of the *ifh1*Δ strain suggest that simply masking the FHA domain is sufficient to enable growth of the *ifh1*Δ strain. Given that Crf1 also has an FHB domain that interacts with the FHA domain of Fhl1, competitively with Ifh1, we tested the effect of Crf1 overexpression on growth of the *ifh1*Δ strain. The *ifh1*Δ strain harboring *IFH1*/*URA3* plasmid was transformed with the high‐copy plasmid expressing wild‐type Crf1 from *CRF1* promoter or *TDH3* promoter, and the transformants were spotted on the 5‐FOA plate. Consistent with the previous reports, expression of Crf1 could enable growth of the *ifh1*Δ strain weakly (*CRF1*p) or strongly (*TDH3*p) (Figure 5A). Next, to examine which region of Crf1 is responsible for this compensation of the growth of *ifh1*Δ strain, high‐copy plasmids expressing various Crf1 deletion mutant proteins from *TDH3* promoter were transformed into the *ifh1*Δ strain harboring *IFH1*/*URA3* plasmid, and the transformants were spotted on the 5‐FOA plate. The results clearly showed that Crf1 mutant proteins containing the 248–407aa region overlapping with the FHB domain could enable growth of the *ifh1*Δ strain to a degree comparable to full‐length Crf1 (wild‐type, Δ2–67, Δ2–127, Δ2–187, Δ2–187, Δ2–247, Δ408–467, and 248–407 in Figure 5B), in other words, N‐terminal 248aa region was dispensable for this activity. To determine whether this growth‐complementing activity of the FHB domain of Crf1 depends on its interaction with the FHA domain, mutations T348A or ST(347,348)AA, whose phosphorylation is important for interaction with Fhl1, were introduced into either the full‐length Crf1 or 248–407 mutant of Crf1, and examined their growth‐complementing activity for the *ifh1*Δ strain. As the result, these mutations did not affect growth‐complementing ability of full‐length Crf1, while they diminished almost, if not all, growth‐complementing ability of 248–407aa region of Crf1 for the *ifh1*Δ strain (Figure S5). These results suggest that the binding of FHB domain of Crf1 to the FHA domain is quite important for its complementing activity for growth of the *ifh1*Δ strain. They also suggest that some secondary, weaker interaction surfaces exist within the deleted regions (2–247aa and/or 408–467aa) that help mask the growth‐inhibitory activity of the FHA domain. As the result of stepwise truncation of Crf1, we have identified 335–406aa region as the minimum region retaining this growth‐complementing activity for the *ifh1*Δ strain (Figure 5C), within this region no function other than binding to FHA domain of Fhl1 has been identified at present.

**FIGURE 6:**
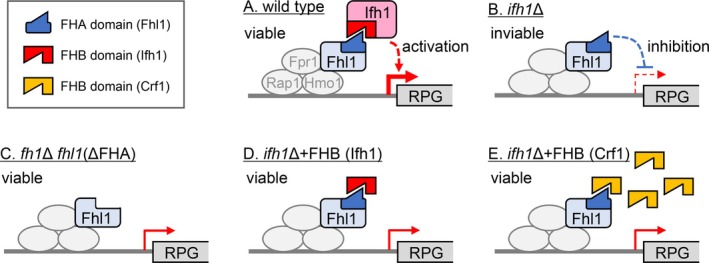
A model for the essential role of Ifh1 in cell growth and the functions of Fhl1 and Crf1 that compensate for the lethality of the *ifh1*Δ mutant. (A) Wild‐type strain. Fhl1 recruits Ifh1 to the promoter of RPGs, thereby promoting cell growth. The precise mechanism underlying this growth‐promoting activity remains unclear. At the same time, Ifh1 masks the growth‐inhibitory activity of the FHA domain of Fhl1. (B) *ifh1*Δ strain. In the absence of Ifh1 due to deletion (or dissociation), the FHA domain of Fhl1 becomes exposed and inhibits cell growth through an unknown mechanism. (C) *ifh1*Δ *fhl1*(ΔFHA) strain. Deletion of the FHA domain from Fhl1 abolishes its growth‐inhibitory activity, resulting in restoration of growth of *ifh1*Δ strain. (D) *ifh1*Δ strain expressing the FHB domain of Ifh1. The FHB domain of Ifh1 suppresses the growth‐inhibitory activity of the FHA domain of Fhl1. (E) *ifh1*Δ strain overexpressing the FHB domain of Crf1. Overexpression of the FHB domain of Crf1 similarly suppresses the growth‐inhibitory activity of the FHA domain of Fhl1, as observed in (D).

Based on the observation in this study, we propose a model for the regulation of cell growth through functional interplay among Fhl1, Ifh1, and Crf1 (Figure 6).

## 3 Discussion

Ifh1 functions as a central regulatory hub that integrates various signals in response to the intracellular and extracellular environments to regulate transcription of RPGs. Although Ifh1 is regarded as essential for yeast growth, previous reports and our study demonstrate that its essentiality is conditional and can be suppressed in certain genetic contexts. By systematically analyzing various Ifh1, Fhl1, and Crf1 mutants, we uncovered mechanisms by which the essential role of Ifh1 can be bypassed, providing new insights into the conventional model of Ifh1 function in RPG transcription, in which Ifh1 acts solely as an indispensable transcriptional activator.

At the start of this study, we investigated which region of Fhl1 is involved in suppressing the growth of *ifh1*Δ strain by expressing various *FHL1* deletion mutants in the *ifh1*Δ*fhl1*Δ strain to examine their ability to enable growth of this strain. The results indicated that the FHA domain plays a central role in the lethality of *ifh1*Δ strain. Expression of wild‐type Fhl1 in an *ifh1*Δ*fhl1*Δ strain strongly inhibited growth, whereas deletion of the FHA domain or even a single amino acid substitution within the FHA domain (S325R or G303A) restored the viability of the *ifh1*Δ strain (Figure 2A,B). The FHA domain is widely conserved in eukaryotic proteins and is found in proteins with diverse biological roles, including transcription factors, RNA‐binding proteins, DNA damage checkpoint regulators, E3 ubiquitin ligases, and metabolic enzymes (Durocher and Jackson 2002). Many FHA domains interact with target molecules by recognizing phosphorylated threonine. The FHA domain of Fhl1 also recognizes phosphorylated threonine residues to interact with its binding factors (T681 of Ifh1 and T347 of Crf1), and this phosphorylation‐dependent interaction contributes to the recruitment of these factors to promoters (Kim and Hahn 2016). Given that expression of Fhl1 in the *ifh1*Δ strain inhibits growth, Fhl1 may recruit factor(s) that repress Ifh1‐independent RPG transcription, or some other cellular function(s) that are crucial for growth, particularly in the absence of Ifh1. Although only Ifh1 and Crf1 have been identified as binding partners of the FHA domain of Fhl1, our findings raise the possibility that additional factor(s) interact with the FHA domain to mediate its growth‐inhibitory activity and that S325R and G303A mutations in the FHA domain may disrupt such interaction(s).

An important finding is that the essential function of Ifh1 is largely confined to its FHB domain, which binds to the FHA domain of Fhl1. Expression of the 601–800aa region of Ifh1, and even a shorter portion narrowed to 678–788aa, enables the growth of the *ifh1*Δ strain (Figures 4A and S3). This result indicates that neither the large N‐terminal portion nor the C‐terminal portion of Ifh1 is required for growth, at least under nutrient‐rich, nonstress conditions, consistent with a previous report (Albert et al. 2016). Although the minimal domain (678–788aa) includes a portion of the transcriptional activation domain of Ifh1 (AD1), which was identified based on its ability to promote reporter gene transcription when fused to the Gal4 DNA‐binding domain (Zhong and Melcher 2010), its contribution to transcriptional promotion in the native Ifh1 context remains unclear. Importantly, mutations that disrupt phosphorylation‐dependent interactions between FHA and FHB domains, such as T681A and ST(680, 681)AA, which are located outside the AD1 region, eliminate the rescue activity of the 678–788aa region and markedly impair most of the rescue activity of the 601–800aa region (Figure S3). Thus, the essential role of Ifh1 is to bind to the FHA domain and neutralize its growth‐inhibitory activity, rather than to activate transcription. The observation that full‐length Ifh1 carrying the same mutations retains its rescue ability implies the existence of secondary, weaker interaction surfaces that help mask the growth‐inhibitory activity of the FHA domain.

Furthermore, we found that overexpression of full‐length Ifh1 enhanced the growth of the *fhl1*Δ strain, indicating that Ifh1 retains growth‐promoting activity in the absence of Fhl1. This effect was significantly reduced by deletion of the N‐terminal 2–200aa region, whereas deletion of the AD1 or AD2 activation domains had no impact (Figure 4B). Notably, overexpression of either full‐length Ifh1 or its N‐terminal region, both of which enhanced the growth of the *fhl1*Δ strain, did not increase RPG transcription (Figure S4). Previous studies showed that artificial recruitment of N‐terminally truncated Ifh1 to the *GAL1* promoter results in weaker transcriptional activation than wild‐type Ifh1 (Martin et al. 2004), and that at least seven lysine residues within the N‐terminal region of Ifh1 are acetylated by Gcn5, negatively regulating Ifh1‐dependent transcription (Downey et al. 2013). Although these findings suggest a role for the N‐terminal region in transcriptional activation, our results indicate that the N‐terminal region of Ifh1 enhances the growth of the *fhl1*Δ strain without significantly stimulating RPG transcription at least under our experimental conditions. These data suggest that Ifh1 possesses a growth‐promoting activity that is mechanistically distinct from transcriptional activation. The molecular mechanism underlying this growth‐promoting activity of Ifh1 remains to be elucidated, including whether Ifh1 functions as a transcriptional activator under more physiologically relevant conditions.

In this study, we demonstrated that the essential role of Ifh1 in growth can be substituted by overexpression of the FHB domain of Crf1. Crf1 is the paralog of Ifh1, and both are thought to have diverged from a common ancestral molecule after WGD (Whole g enome duplication) process. Crf1 has been described as a transcriptional repressor that competes with Ifh1; however, its repressive function varies among different strains, even within the same *S. cerevisiae* . An inhibitory effect on RPG transcription was observed in the TB50 strain, but not in the W303‐1A strain (Rudra et al. 2007). In contrast, the ability of Crf1 to rescue the lethality of *ifh1*Δ strain, when overexpressed, is observed at least among two different strains W303‐1A and BY4741 (Zencir et al. 2020; Figure 5), the latter is closely related to the S288C and TB50 strains (Boeckstaens et al. 2014). As an interpretation of this rescue activity, Crf1 was proposed to be a weak (Kumar et al. 2024) or unregulatable (Zencir et al. 2020) transcriptional activator compared to Ifh1, and not a repressor, as previously thought. Indeed, transcription of target genes of Crf1 such as *HMO1* and *UTP22* has been reduced in the *crf1*Δ strain (Kumar et al. 2024). On the other hand, we localized the rescue activity for *ifh1*Δ to the 335–406aa region of Crf1, corresponding to its FHB domain, and no transcription‐promoting activity has been reported within this region. Importantly, mutations that disrupt phosphorylation‐dependent interactions between the FHA domain and FHB of Crf1, T348A, and ST(347,348)AA, almost eliminated the rescue activity of the 248–407aa regions (Figure S5). These results support the idea that Crf1 serves as an anti‐repressor, rather than acting as a canonical activator in the rescue of the *ifh1*Δ strain. In this model, Crf1 is hypothesized to temporarily suppress the growth‐inhibitory effect of the FHA domain when Ifh1 is unavailable, suggesting its role in maintaining transcriptional responsiveness under conditions unfavorable for growth. This model is consistent with the reports that the *crf1*Δ strains exhibit reduced fitness during prolonged rapamycin exposure (Kumar et al. 2024). While our results do not negate the transcription‐promoting ability of Crf1, we speculate that the mechanism for the transcriptional activation of promoters that bind Fhl1 and Crf1 likely involves the release of transcriptional repression by the FHA domain.

Based on these data, we propose a revised model for the interaction network formed by Fhl1, Ifh1, and Crf1 in regulation of RPG transcription. Under nutrient‐rich conditions, the FHA domain of Fhl1 recruits Ifh1 to RPG promoters to promote growth via transcriptional activation or other unknown mechanisms, whereas Ifh1 masks the latent growth‐inhibitory activity of the FHA domain. During starvation or stress, Ifh1 dissociates from the FHA domain, leading to reduced transcription and simultaneous growth inhibition via the exposed FHA domain. This mutually controlled interaction between Fhl1 and Ifh1 may function by actively promoting growth while masking the growth‐inhibitory effect of Fhl1 under favorable growth conditions. Conversely, under unfavorable growth conditions, the FHA domain tightly restricts transcription and cell proliferation, thereby conserving energy and cellular resources. Crf1 is recruited under these conditions and prevents inappropriate reassociation of Ifh1, thereby repressing transcription (in a strain‐specific manner), while simultaneously reducing damage from prolonged exposure to the FHA‐mediated inhibitory state and maintaining transcriptional responsiveness under unfavorable conditions until conditions improve. Thus, Fhl1, Ifh1, and Crf1 possess dual functions, and each factor mutually regulates the function of its binding partner in response to environmental conditions. This model provides a new conceptual basis for understanding how ribosomal biogenesis coordinates with environmental and metabolic signals, integrating growth control and transcriptional regulation at a previously unrecognized level.

## 4 Experimental Procedures

### 4.1 Yeast Strains, Plasmids

Standard techniques were used for the growth and transformation of yeast. The yeast strains used in this study are listed in Table S1. Detailed information regarding construction of each yeast strain and plasmid are available on request. The yeast culture conditions for each experiment are described in the figure legends. A diploid *IFH1*/*ifh1*Δ cells (24172) was obtained from Euroscarf (Frankfurt, Germany). A diploid strain (BY4502), which is consist of W303 strains, was provided by the National Bio‐Resource Project (NBRP), Japan (JPNBRP202225).

### 4.2 Immunoblot Analysis

Yeast cell extract was prepared according to a published protocol (Kushnirov 2000). Proteins were detected using an anti‐FLAG tag monoclonal antibody (Sigma‐Aldrich, St. Louis, MO, USA; M2). Secondary antibodies against mouse was obtained from Proteintech (Rosemont, IL, USA; RGAM001).

### 4.3 RT‐qPCR

Quantitation of mRNA of yeast genes was conducted by RT‐qPCR methods. cDNA was synthesized from prepared RNA using PrimeScript RT Master Mix (Perfect Real Time) (Takara bio, Kusatsu, Japan) according to the manufacturer's protocol. qPCR was conducted using the prepared cDNA as template and KAPA SYBR FAST qPCR Kit Master Mix (2×) (KAPA Biosystems, Wilmington, MA, USA).

An artificial intelligence‐based tool (ChatGPT, OpenAI) was used solely to assist with English sentence structure and wording; no scientific content, data interpretation, or conclusions were generated by this tool.

## Author Contributions

**Naoki Shimamura:** data curation, investigation, visualization, writing – review and editing. All authors have read and approved the final version of the manuscript and agree to its submission to Genes to Cells. **Koji Kasahara:** conceptualization, data curation, funding acquisition, investigation, project administration, resources, supervision, validation, visualization, writing – original draft.

## Funding

This work was supported by Japan Society for the Promotion of Science (JP20K05965 and JP23K05153).

## Ethics Statement

The authors have nothing to report.

## Conflicts of Interest

The authors declare no conflicts of interest.

## Supporting information


**Figure S1:** Complementation of slow growth of the *fhl1*Δ mutant by various Fhl1 mutant proteins. (A) Predicted aligned error (PAE) for Fhl1 (quoted from SDG: *Saccharomyces* GENOME DATABASE; https://www.yeastgenome.org/) by AlphaFold 3. The PAE plot predicted three domains that did not interact with each other (Domains 1–3). Domains 1 and 2 correspond to the FHA and FH domains, respectively, whereas Domain 3 is not well‐characterized. (B) Effect of deletion of various regions within Fhl1 on growth was examined. Ten‐fold dilutions of the *fhl1*Δ strain expressing various Fhl1 deletion mutants with FLAG×3 tag at C‐terminus, illustrated to the left of the images, were spotted on the three SD‐Leu plates, and were grown at 20°C, 30°C, and 37°C for 3 days. Dark gray squares represent the forkhead‐associated (FHA) domain, the FH domain, and a third domain predicted by AlphaFold 3, respectively, as shown in (A). (C) Immunoblotting for Fhl1 mutant proteins expressed in (A). Cell extracts prepared from mid‐log phase cells grown at 30°C in SD‐Leu media were subjected to SDS‐PAGE and immunoblotting. The Fhl1 protein was detected using an anti‐FLAG tag antibody.
**Figure S2:** Mutations of the FHA domain impair the growth‐inhibitory activity of Fhl1 against the *ifh1*Δ strain. (A) Immunoblotting of wild‐type, ΔFHA, and S325R Fhl1 proteins. These Fhl1 proteins were expressed from low‐copy (lanes 1, 5, and 9) or high‐copy (lanes 3, 7, and 11) plasmids on the BY4741 background, or low‐copy on the W303‐1A background (lanes 4, 8, and 12), and were detected using an anti‐FLAG tag antibody. Lanes 2, 6, and 10 show expression of the Fhl1 mutants encoded by the *FHL1* gene integrated at the chromosomal *FHL1* locus in BY4741. (B) High‐copy plasmid expressing wild‐type, ΔFHA, or S325R Fhl1 proteins used in A were transformed into the *ifh1*Δ*fhl1*Δ strain. These transformants were spread on the SD‐Leu medium and grown at 30°C. Images were acquired 11 days (upper panel) or 16 days (lower panel) after transformation. (C) The *ifh1*Δ*fhl1*Δ strain harboring *IFH1/URA3* plasmid was transformed with the plasmids used in B and a plasmid expressing ΔFH (Δ440‐567) Fhl1. These transformants were spotted on the medium with or without 5‐FOA at three dilutions and grown at 30°C for 5 or 15 days.
**Figure S3:** Complementation of growth of *ifh1*Δ strain requires binding activity of FHB domain of Ifh1 to FHA domain of Fhl1. Effect of Ifh1 on the growth of *ifh1*Δ strain was examined. Low‐copy plasmids expressing wild‐type Ifh1, full‐length Ifh1 containing T681A or ST(680, 681)AA mutation, the 601‐800aa fragment, the 601‐800aa fragment containing T681A or ST(680, 681)AA mutation, and Δ678‐715 mutant from own promoter were transformed into the *ifh1*Δ strain. Similarly, high‐copy plasmids expressing the smallest region of the FHB domain (678–788aa), and 678–788aa fragment containing T681A or ST(680, 681)AA mutation from *TDH3* promoter, which is a very strong promoter (overexpression) were transformed into the *ifh1*Δ strain. These transformants were spotted on medium with or without 5‐FOA at three dilutions and grown for 3, and 10 days at 30°C as described in Figure 4A.
**Figure S4:** Effect of overexpression of various Ifh1 mutant proteins on RPG transcription in the *fhl1*Δ strain. The *fhl1*Δ strains overexpressing the Ifh1 mutant proteins used in Figure 4 were cultured in 50 mL SD‐Leu medium at 30°C to an OD600 of approximately 0.6–0.7. Total RNA was isolated from the cultures, and reverse transcription quantitative PCR (RT‐qPCR) was performed to quantify mRNA levels of *RPS5*, *RPL25*, *RPS25A*, and *PGK1*. RPG mRNA levels were normalized to *PGK1* mRNA and are shown as bar graphs. Experiments were performed nine times (*n* = 9), and data represent the mean ± SD. Statistical significance was evaluated by Student's *t*‐test comparing strains expressing Ifh1 mutants with the empty plasmid control (*p < 0.05, **p < 0.01).
**Figure S5:** Complementation of growth of *fhl1*Δ strain by overexpression of various Crf1 mutant proteins
Plasmids expressing the full‐length or the 248–407aa region of Crf1 with or without mutations (T348A or ST(347,348)AA) were transformed into *ifh1*Δ strain harboring *IFH1/URA3* plasmid. These transformants were spotted on the 5‐FOA plate and grown for 3, and 10 days at 30°C as described in Figure 5B.

**Table S1:** gtc70109‐sup‐0002‐TableS1.xlsx. *S. cerevisiae* strains used in this study.

## Data Availability

All data supporting the findings of this study are available within the article and its Supporting Information.
