# Supplementary material for: Regulation of the Growth‐Inhibitory Activity of Fhl1 via Interaction With Ifh1 and Crf1 at the Ribosomal Protein Gene Promoters in Saccharomyces cerevisiae
Source: Genes Cells. 2026 Apr 7;31(3):e70109. doi: 10.1111/gtc.70109 (PMC13054639; doi:10.1111/gtc.70109)
Supplement: Supplementary file 1 — Figure S1: Complementation of slow growth of the fhl1Δ mutant by various Fhl1 mutant proteins. (A) Predicted aligned error (PAE) for Fhl1 (quoted from SDG: Saccharomyces GENOME DATABASE; https://www.yeastgenome.org/) by AlphaFold 3. The PAE plot predicted three domains that did not interact with each other (Domains 1–3). Domains 1 and 2 correspond to the FHA and FH domains, respectively, whereas Domain 3 is not well‐characterized. (B) Effect of deletion of various regions within Fhl1 on growth was examined. Ten‐fold dilutions of the fhl1Δ strain expressing various Fhl1 deletion mutants with FLAG×3 tag at C‐terminus, illustrated to the left of the images, were spotted on the three SD‐Leu plates, and were grown at 20°C, 30°C, and 37°C for 3 days. Dark gray squares represent the forkhead‐associated (FHA) domain, the FH domain, and a third domain predicted by AlphaFold 3, respectively, as shown in (A). (C) Immunoblotting for Fhl1 mutant proteins expressed in (A). Cell extracts prepared from mid‐log phase cells grown at 30°C in SD‐Leu media were subjected to SDS‐PAGE and immunoblotting. The Fhl1 protein was detected using an anti‐FLAG tag antibody. Figure S2: Mutations of the FHA domain impair the growth‐inhibitory activity of Fhl1 against the ifh1Δ strain. (A) Immunoblotting of wild‐type, ΔFHA, and S325R Fhl1 proteins. These Fhl1 proteins were expressed from low‐copy (lanes 1, 5, and 9) or high‐copy (lanes 3, 7, and 11) plasmids on the BY4741 background, or low‐copy on the W303‐1A background (lanes 4, 8, and 12), and were detected using an anti‐FLAG tag antibody. Lanes 2, 6, and 10 show expression of the Fhl1 mutants encoded by the FHL1 gene integrated at the chromosomal FHL1 locus in BY4741. (B) High‐copy plasmid expressing wild‐type, ΔFHA, or S325R Fhl1 proteins used in A were transformed into the ifh1Δfhl1Δ strain. These transformants were spread on the SD‐Leu medium and grown at 30°C. Images were acquired 11 days (upper panel) or 16 days (lower panel) after transfor [file GTC-31-0-s002.pptx]

## Slide 1
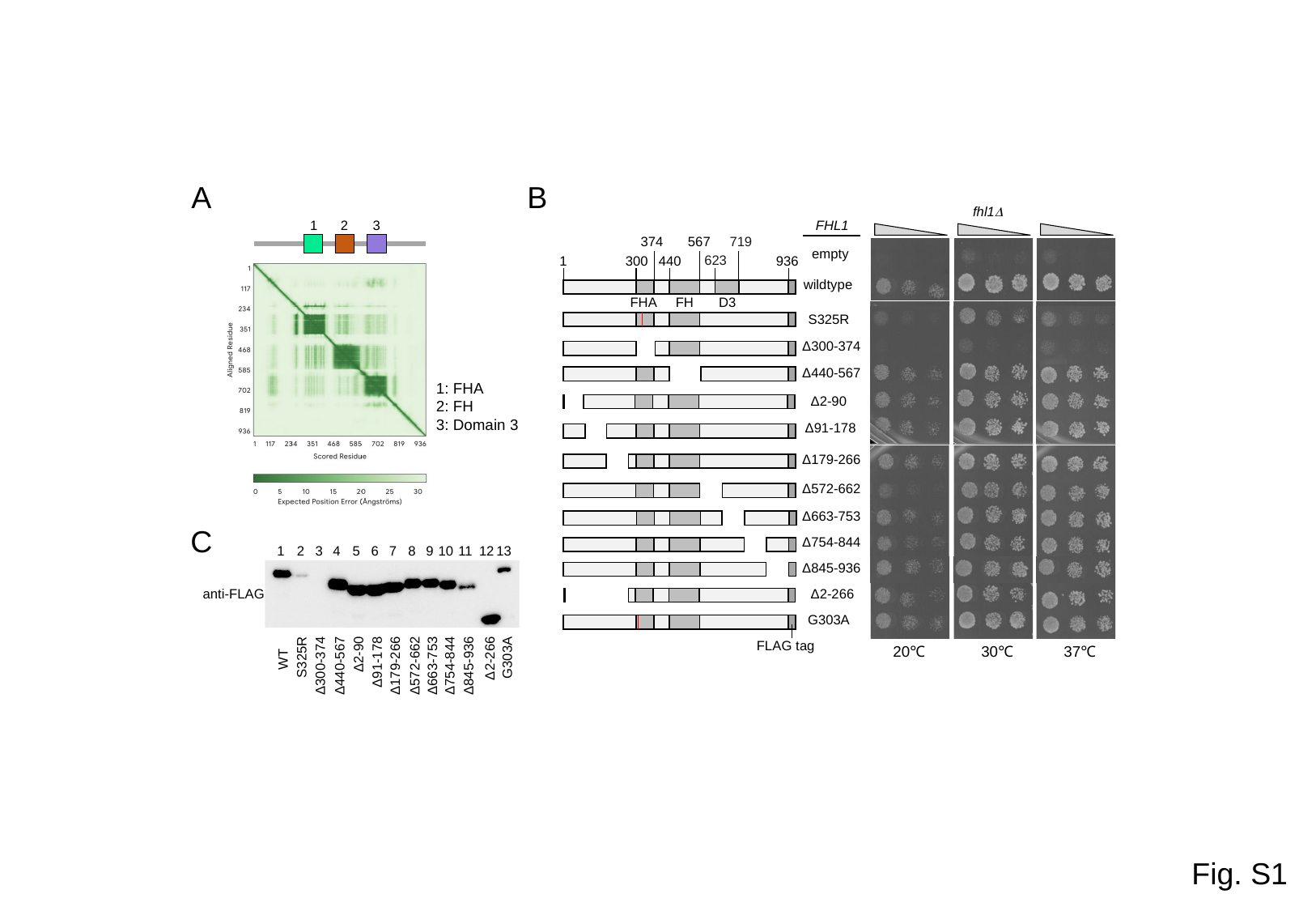

A
B
fhl1Δ
FHL1
1
2
3
374
567
719
empty
623
1
300
440
936
wildtype
FHA
FH
D3
S325R
Δ300-374
Δ440-567
1: FHA
2: FH
3: Domain 3
Δ2-90
Δ91-178
Δ179-266
Δ572-662
Δ663-753
C
Δ754-844
1
2
3
4
5
6
7
8
9
10
11
12
13
Δ845-936
anti-FLAG
Δ2-266
G303A
FLAG tag
30℃
37℃
20℃
Δ2-90
WT
S325R
Δ2-266
G303A
Δ300-374
Δ572-662
Δ440-567
Δ91-178
Δ663-753
Δ754-844
Δ845-936
Δ179-266
Fig. S1

## Slide 2
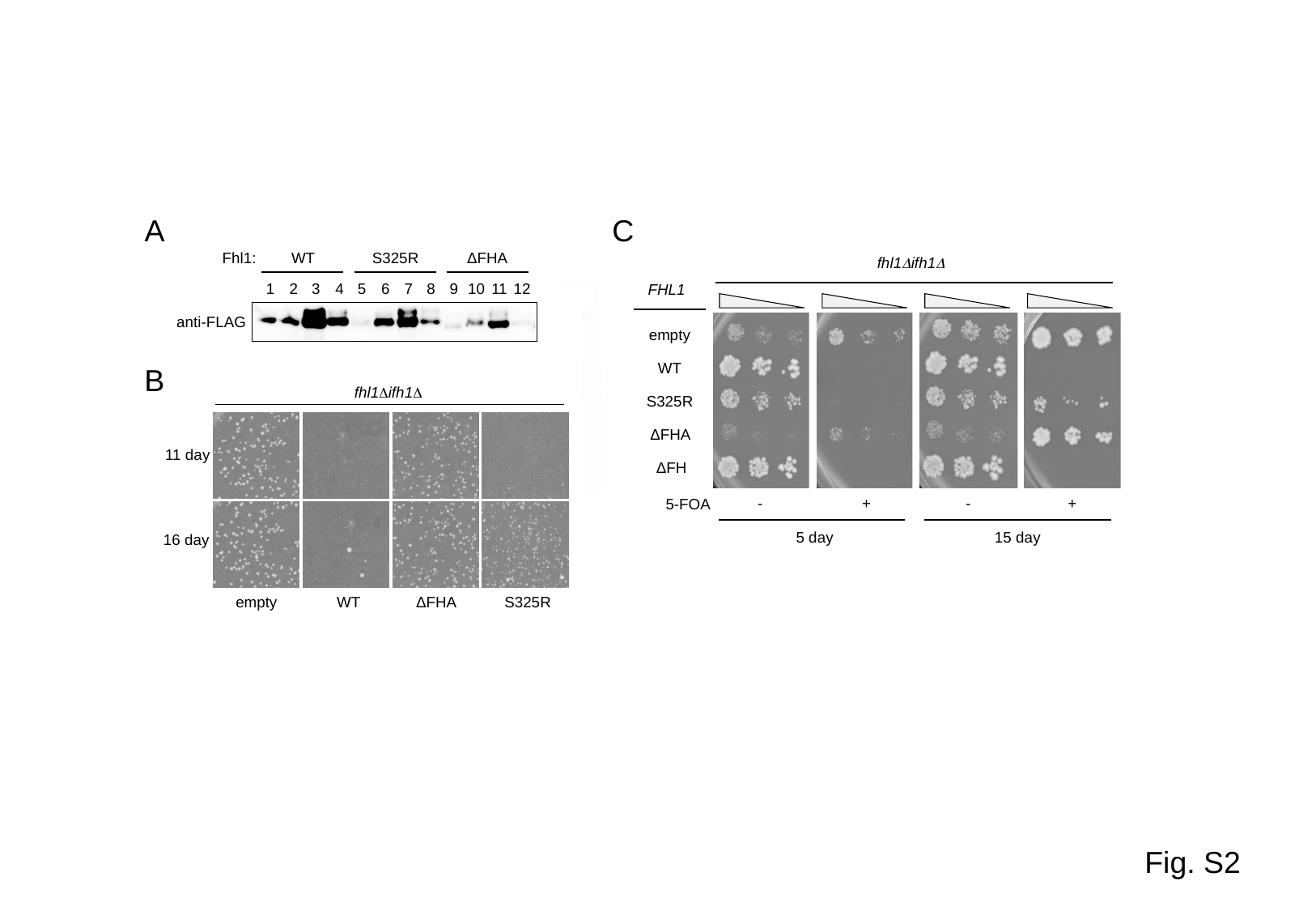

A
C
Fhl1:
WT
S325R
ΔFHA
fhl1Δifh1Δ
1
2
3
4
5
6
7
8
9
10
11
12
FHL1
anti-FLAG
empty
WT
B
fhl1Δifh1Δ
S325R
ΔFHA
11 day
ΔFH
-
+
-
+
5-FOA
5 day
15 day
16 day
S325R
empty
WT
ΔFHA
Fig. S2

## Slide 3
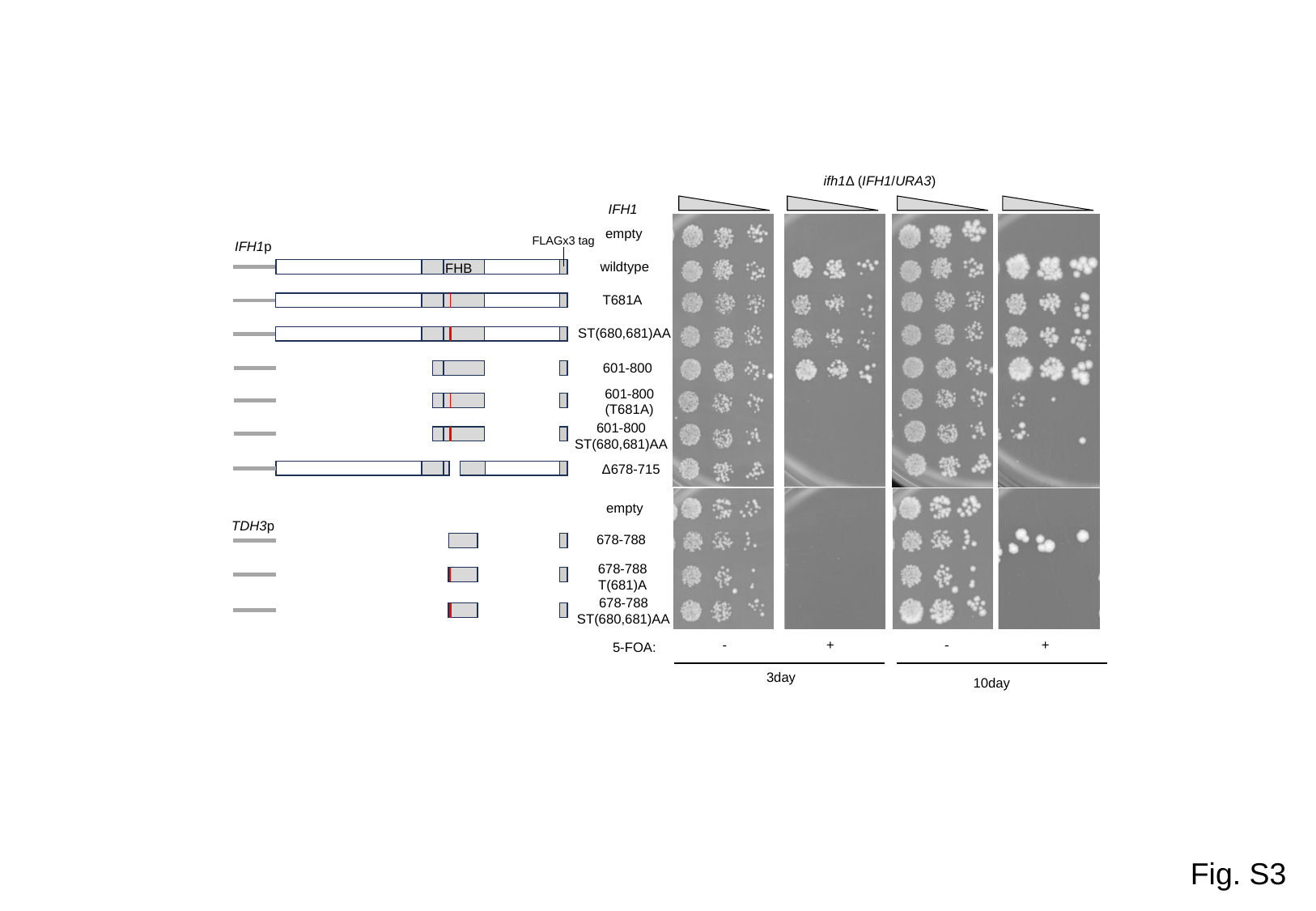

ifh1Δ (IFH1/URA3)
IFH1
empty
FLAGx3 tag
IFH1p
wildtype
FHB
T681A
ST(680,681)AA
601-800
601-800
(T681A)
601-800
ST(680,681)AA
Δ678-715
empty
TDH3p
678-788
678-788
T(681)A
678-788 ST(680,681)AA
-
+
-
+
5-FOA:
3day
10day
Fig. S3

## Slide 4
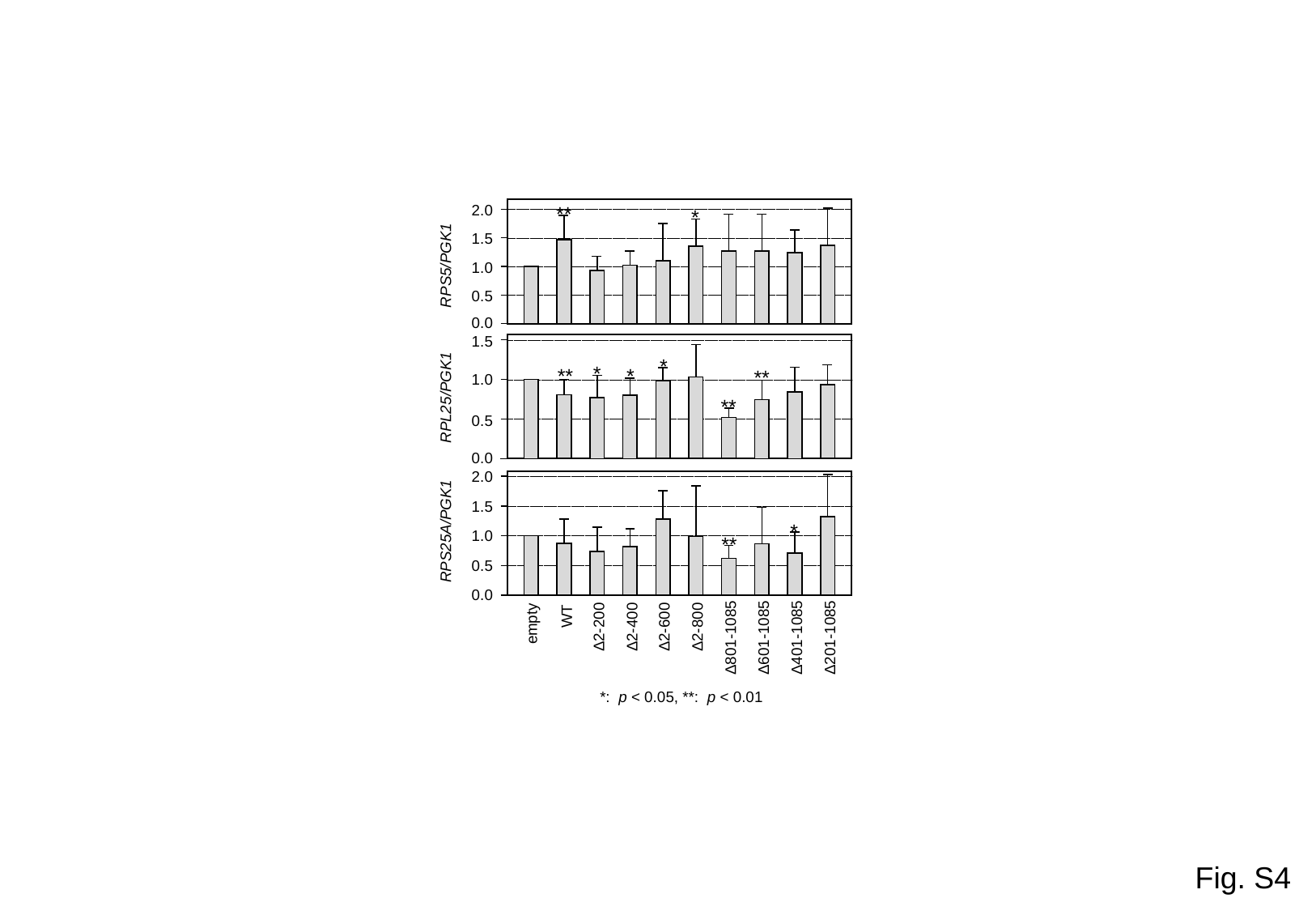

2.0
**
*
1.5
RPS5/PGK1
1.0
0.5
0.0
1.5
*
*
*
**
**
1.0
RPL25/PGK1
**
0.5
0.0
2.0
1.5
*
RPS25A/PGK1
1.0
**
0.5
0.0
WT
empty
Δ2-200
Δ2-400
Δ2-600
Δ2-800
Δ801-1085
Δ601-1085
Δ401-1085
Δ201-1085
*: p < 0.05, **: p < 0.01
Fig. S4

## Slide 5
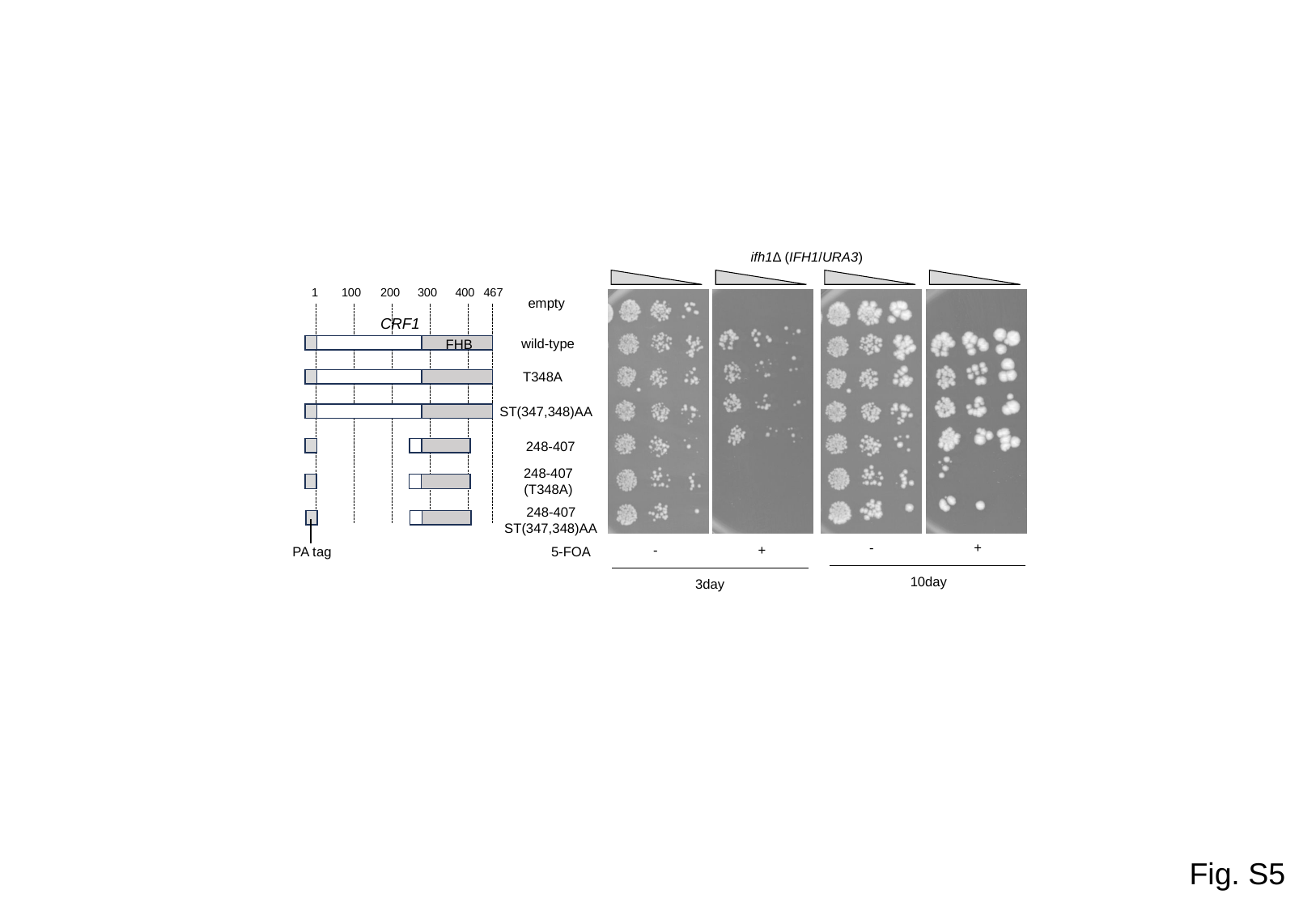

ifh1Δ (IFH1/URA3)
1
100
200
300
400
467
empty
CRF1
wild-type
FHB
T348A
ST(347,348)AA
248-407
248-407
(T348A)
248-407
ST(347,348)AA
-
+
-
+
PA tag
5-FOA
10day
3day
Fig. S5
